# Sequence analysis, expression profiles and function of thioredoxin 2 and thioredoxin reductase 1 in resistance to nucleopolyhedrovirus in *Helicoverpa armigera*

**DOI:** 10.1038/srep15531

**Published:** 2015-10-27

**Authors:** Songdou Zhang, Zhen Li, Xiaoge Nian, Fengming Wu, Zhongjian Shen, Boyu Zhang, Qingwen Zhang, Xiaoxia Liu

**Affiliations:** 1 Department of Entomology, China Agricultural University, Beijing, 100193, China

## Abstract

The thioredoxin system, including NADPH, thioredoxin (Trx), and thioredoxin reductase (TrxR), plays significant roles in maintaining intracellular redox homeostasis and protecting organisms against oxidative damage. In this study, the characteristics and functions of *H. armigera HaTrx2* and *HaTrxR1* were identified. Sequence analysis showed that *HaTrx2* and *HaTrxR1* were both highly conserved and shared high sequence identity with other insect counterparts. The mRNA of *HaTrx2* was expressed the highest in 5th instar 96 h and was mainly detected in heads and epidermis. The expression of *HaTrxR1* was highly concentrated in 5th instar 72 h and 96 h, and higher in malpighian tube, midgut and hemocyte than other examined tissues. *HaTrx2* and *HaTrxR1* were markedly induced by various types of stress. HaTrx2- or HaTrxR1-knockdown increased ROS production in hemocytes and also increased the lipid damage in NPV infected *H. armigera* larvae. Furthermore, interference with expression of *HaTrx2* or *HaTrxR1* transcripts in *H. armigera* larvae resulted in increased sensitivity to NPV infection and shortened LT_50_ values. Our findings indicated that *HaTrx2* and *HaTrxR1* contribute to the susceptibility of *H. armigera* to NPV and also provided the theoretical basis for the in-depth study of insect thioredoxin system.

Oxidative stress, which can damage the balance of strong oxidants and antioxidants, is a negative effect produced by reactive oxygen species (ROS) in living organisms and is one of the important factors that cause aging and disease^1^,^2^. ROS, including hydrogen peroxide, superoxide anions and hydroxyl radicals, can be induced by various adverse factors, leading to oxidative damage to biological macromolecules^3^. To protect against these toxic oxygen intermediates caused by ROS, oxygenic organisms have developed a battery of protective enzymes, including the antioxidase system, and non-enzymatic systems to maintain redox homeostasis by scavenging excessive ROS^4^,^5^. The thioredoxin system, comprising NADPH, thioredoxin (Trx), and thioredoxin reductase (TrxR), is one of these systems that participate in redox-regulatory processes in cells^6^.

The Trxs, which contain a highly conserved Cyc-Gly-Pro-Cys (CGPC) active site motif 7, are the family of small reductases (approximately 12 kDa in size) that are ubiquitously distributed from Archaea to man^6^. The Trxs, which were first identified in *Escherichia coli* as an electron donor for ribonucleotide reductase^8^, play numerous roles in resisting oxidative stresses, promoting cell growth, regulating cell apoptosis and transcriptional regulation in many organisms^9^. Oxidized Trxs present a disulfide bond, while reduced Trxs exhibit a thiol group. The reversible thiol-disulfide transformation reactions are rapid and ideally suited to regulate the functions of proteins^4^. It is generally believed that the antioxidant effect of Trxs are mainly manifested in two aspects: first, Trxs can serve as electron donors for peroxidases to cope with ROS and, thus, to reduce lipid peroxidation, DNA damage and protein inactivation; second, as a disulfide reductase of intracellular proteins, Trxs can reduce the disulfide bonds of many proteins (such as kinases, phosphatases and transcription factors) to restore physiological function^10^.

Trxs have been widely studied in mammals^11^,^12^, plants^13^,^14^, and bacteria^15^,^16^ because of their essential roles in protection against oxidative stress, whereas reports focusing on Trxs in insects are limited. In *Drosophila*, three Trx genes (Trx1, Trx2, and TrxT) have been identified^17^,^18^,^19^, and the loss of Trx-2 promoted the expression of other antioxidant genes and exacerbated oxidative stress-dependent phenotypes^20^. In *Bombyx mori*, *BmTrx* has been shown to protect against oxidative stress caused by extreme temperatures and microbial infection^21^. In *Apis mellifera*, three Trxs have been identified: *AmTrx1*, which is located in the mitochondrion, *AmTrx2*, which is a putative ortholog of Drosophila Trx2 and may play a vital role in redox homeostasis, and *AmTrx3*^22^. In *Apis cerana cerana*, some Trxs, including *AccTrx-like1*^23^, *AccTrx2*^24^ and *AccTrx1*^25^, have been demonstrated to participate in antioxidant defense. All of the above studies suggest that Trxs play a major role in maintaining redox homeostasis and resisting adverse circumstances in insects.

TrxRs are homodimeric flavoproteins that belong to the pyridine nucleotide-disulfide oxidoreductase family and can catalyze the natural substrates of thioredoxin^26^. There are two forms of TrxRs in different organisms: low molecular weight (MW) TrxRs, of approximately 35 kDa, which are mainly found in bacteria, plants and parasites; and high MW TrxRs, of approximately 55 kDa, which are mainly found in higher eukaryotes^27^. The N-terminus of mammalian TrxRs possesses a redox catalytic site structure consisting of -Cyc-Val-Asn-Gly-Cys- (CVNVGC), and the C-terminus exhibits an extension redox active site sequence of -Gly-Cys-Sec-Gly- (GCUG)^28^,^29^, while the C-terminal conserved sequence is -Cys-Cys-Ser- (CCS) in insects^30^. TrxRs can transfer reducing equivalents from NADPH to thioredoxin; the electron transfer path is from NADPH to FAD, then to N-terminal redox active sites, followed by the C-terminal active motifs, and finally to Trxs^28^. The physiological roles of TrxRs have been widely studied in mammals, including their functions in redox homeostasis and antioxidant defense^31^, regulating cell growth and inhibiting cell apoptosis^32^, and controlling early embryonic development^33^. There have been some reports about the use of Trx and TrxR as targets of cancer therapy^34^,^35^.

In contrast to the many studies addressing TrxRs in mammals, knowledge of TrxRs in insects is lacking. In *Drosophila*, two TrxRs have been identified: *TrxR-1*, which encodes three splice variants (one mitochondrial and two cytoplasmic forms), and *TrxR-2*, which encodes a protein with a potential targeting peptide^36^. The *TrxR-1* null mutant of *D. melanogaster* leads to death at the end of the second larval instar^37^, and both cytosolic and mitochondrial *TrxR-1* forms have been shown to be necessary for survival^36^. In *Anopheles gambiae*, *TrxR-1*, which occurs in three splice variants, shares 69% sequence identity with *D. melanogaster TrxR-1* and possesses a conserved Cys-Cys active motif in its C-terminal extension^30^. In *A. mellifera*, only one TrxR gene has been identified, which exhibits two putative splice variants, but it does not appear that they encode the mitochondrial variant^22^. In *A. cerana cerana*, *AccTrxR1* was shown to be induced by ultraviolet light (UV) and heat (37 °C) and to be involved in protection against oxidant stress^38^. In *Chironomus riparius*, the transcription of *CrTrxR1* was found to be up-regulated after paraquat and cadmium chloride exposure and is considered to be a biomarker of oxidative stress induced by environmental contaminants^39^.

The cotton bollworm (*Helicoverpa armigera*) is one of the lepidopteran pests that cause the most damage, resulting in enormous economic losses in the cotton, corn, vegetable and other crop industries throughout Asia^40^. Although its population has decreased since the introduction of Bt-cotton in China in 1997, the control of this pest is a longstanding problem due to its ability to develop insecticide resistance^40^,^41^. The genes of the thioredoxin system are being considered as targets for the treatment of inflammation or cancer in humans^42^,^43^, and another antioxidant gene (thioredoxin peroxidase) was shown to be involved in resistance to the biocontrol fungus *Nomuraea rileyi* in *Spodoptera litura*^44^. We hypothesize that Trx and TrxR can help to resist the infection of pathogenic microorganism in insect. To elucidate the functions of thioredoxin system genes in *H. armigera*, we investigated their spatio-temporal distribution and evaluated their transcript levels after various types of stress treatments, including temperatures of 0 °C and 37 °C, UV, mechanical injury, *E. coli* exposure, *Metarhizium anisopliae* exposure, and nucleopolyhedrovirus (NPV) infection. Furthermore, ROS generation and lipid peroxidation in HaTrx2- or HaTrxR1-knockdown larvae and normal larvae were measured. Finally, RNA interference (RNAi) technology was used to study these two genes involved in resistance to NPV. Our results will contribute to further studies on Trx and TrxR in Insecta and will aid in the development of novel insecticides targeting Trx and TrxR.

## Results

### Sequence analysis of *HaTrx2* and *HaTrxR1*

**Figure 1:**
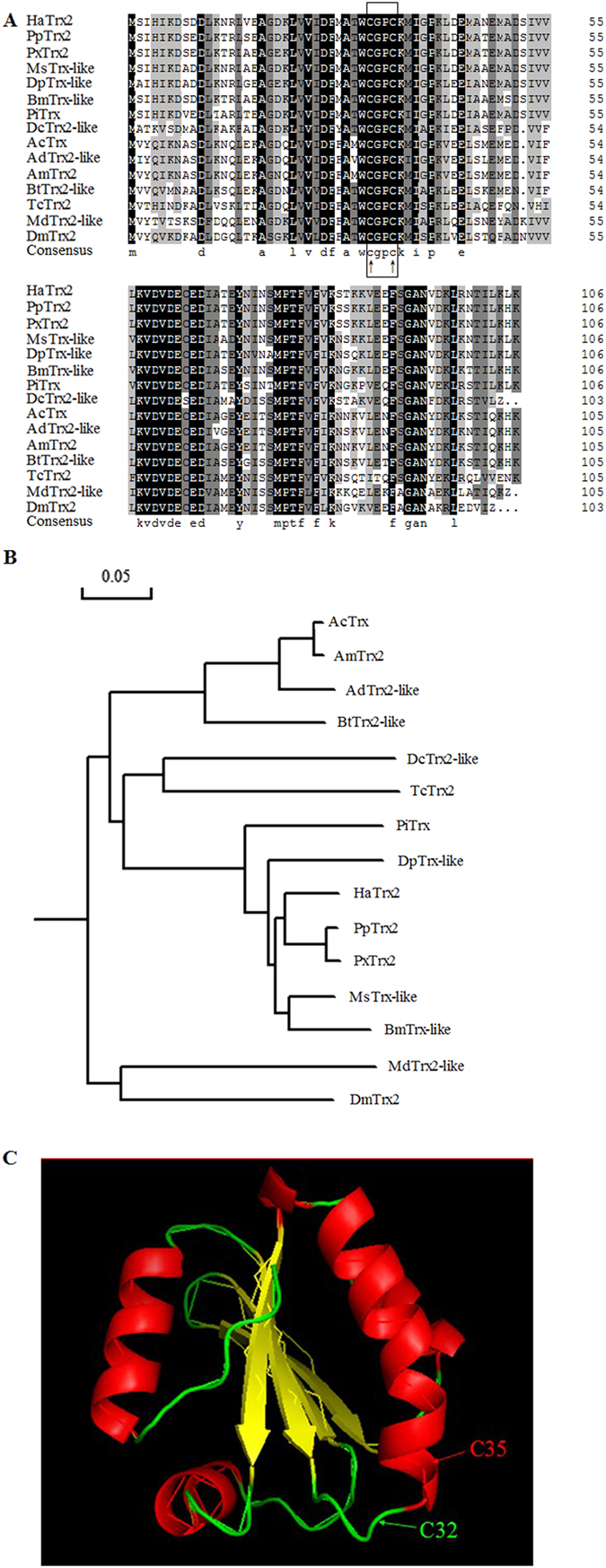
Sequence characterization of Trx from various species and the predicted tertiary structure of *HaTrx2*. (**A**) Multiple alignments of the amino acid sequence of *HaTrx2* with homologs from other insect species. Black represents 100% identity, gray represents 75% identity and white represents <75% identity. The conserved CGPC motif is boxed and the active sites are marked by ↑. *HaTrx2* (*Helicoverpa armigera*, AGC39043.1), *PpTrx2* (*Papilio polytes*, BAM19091.1), *PxTrx2* (*Papilio xuthus*, BAM17831.1), *MsTrx-like* (*Manduca sexta*, AAF16695.1), *DpTrx-like* (*Danaus plexippus*, EHJ64037.1), *BmTrx-like* (*Bombyx mori*, NP_001091804.1), *PiTrx* (*Plodia interpunctella*, CBW45298.1), *DcTrx2-like* (*Diaphorina citri*, XP_008485323.1), *AcTrx* (*Apis cerana*, AGF33352.1), *AdTrx2-like* (*Apis dorsata*, XP_006608786.1), *AmTrx2* (*Apis mellifera*, XP_003250408.1), *BtTrx2-like* (*Bombus terrestris*, XP_003396074.1), *TcTrx2* (*Tribolium castaneum*, XP_967987.1), *MdTrx2-like* (*Musca domestica*, XP_005177432.1), *DmTrx2* (*Drosophila melanogaster*, NP_523526.1). The same as below. (**B**) Phylogenetic tree analysis of *HaTrx2* and its homologs in insects. (**C**) Predicted tertiary structure of *HaTrx2*. The SWISS-MODEL server and PyMOL software were used to construct the tertiary structure, and the active sites (Cys^32^ and Cys^35^) are marked.

Sequence analysis showed that the full-length cDNA of *HaTrx2* was 800 bp, including a 321 bp open reading frame (ORF) and encoding a deduced polypeptide of 107 amino acids with a predicted molecular weight of 12.03 kDa and a *pI* of 4.82. Multiple alignment analysis of the amino acid sequence showed that *HaTrx2* shared high amino acid identity (61%–92%) with Trx sequences from other selected insect species. The active site sequence CGPC was found in the N-terminal portion of the *HaTrx2* sequence and was highly conserved among all of the selected insect species (Fig. 1A). As shown in Fig. 1B, phylogenetic analysis revealed that *HaTrx2* was most closely related to the *PpTrx2* homologue (*Papilio polytes*, BAM19091.1) and *PxTrx2* homologue (*Papilio xuthus*, BAM17831.1), consistent with the evolutionary relationships predicted from the multiple alignment of amino acid sequences. The potential tertiary protein structure of *HaTrx2* was constructed with the SWISS-MODEL server and PyMOL-v1.3r1 software, and the cysteines (Cys^32^ and Cys^35^) in the conserved redox active motif were identified (Fig. 1C).

**Figure 2:**
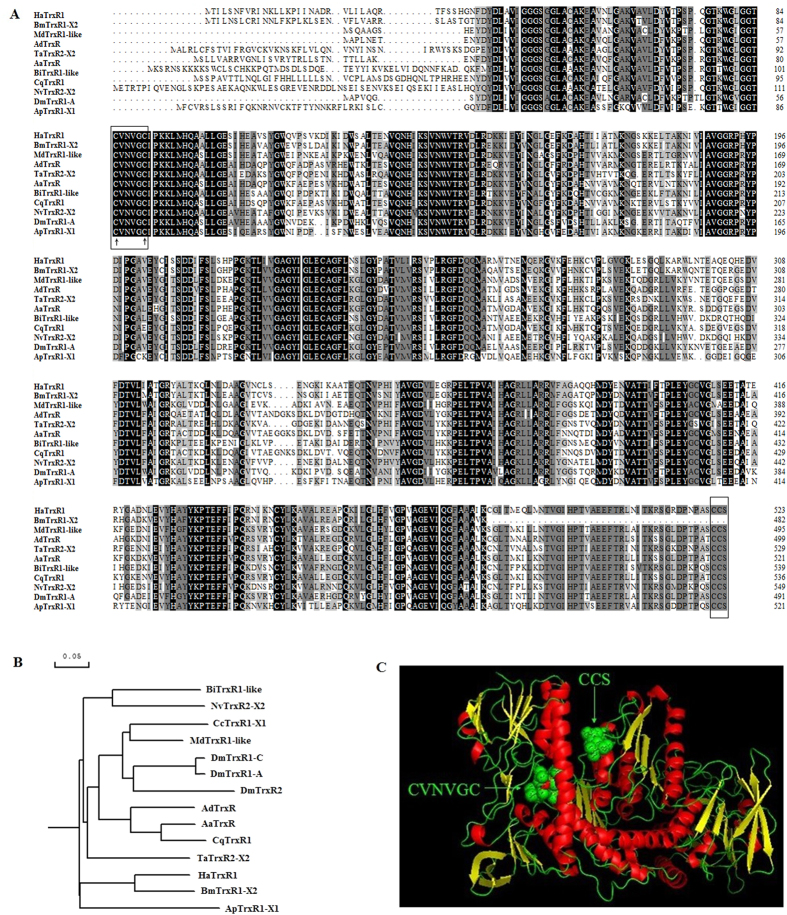
Sequence characterization of TrxR from various species and the predicted tertiary structure of *HaTrxR1*. (**A**) Multiple alignments of the amino acid sequences of *HaTrxR1* with homologs from other insect species. Black represents 100% identity, gray represents 75% identity and white represents <75% identity. The conserved CVNVGC motif and CCS motif are boxed and the active sites are marked by ↑. *HaTrxR1* (*Helicoverpa armigera*, KM658552), *BmTrxR1-X2* (*Bombyx mori*, XP_004921588.1), *MdTrxR1-like* (*Musca domestica*, NP_001273801.1), *AdTrxR* (*Anopheles darlingi*, ETN61621.1), *TaTrxR2-X2* (*Tribolium castaneum*, XP_008191173.1), *AaTrxR* (*Aedes aegypti*, XP_001662666.1), *BiTrxR1-like* (*Bombus impatiens*, XP_003485381.1), *CqTrxR1* (*Culex quinquefasciatus*, XP_001847793.1), *NvTrxR2-X2* (*Nasonia vitripennis*, XP_008202131.1), *DmTrxR1-A* (*Drosophila melanogaster*, NP_727252.1), *ApTrxR1-X1* (*Acyrthosiphon pisum*, XP_008179444.1). The same as below. (**B**) Phylogenetic tree analysis of *HaTrxR1* and its homologs in insects. *CcTrxR1-X1* (*Ceratitis capitata*, XP_004518825.1), *DmTrxR1-C* (*Drosophila melanogaster*, NP_727252.1), *DmTrxR2* (*Drosophila melanogaster*, NP_524216.1). (**C**) Predicted tertiary structure of *HaTrxR1*. SWISS-MODEL server and PyMOL software were used to build the tertiary structure and the conserved motifs (CVNVGC and CCS) were marked.

The ORF of *HaTrxR1* was 1572 bp, encoding a polypeptide of 523 amino acids residues with a predicted molecular weight of 57.16 kDa and a theoretical *pI* of 7.57. Multiple sequence alignment revealed that *HaTrxR1* shared 83% identity with *BmTrxR1-X2* and 64%–71% identity with TrxR sequences from other selected insect species. The active site sequence CVNVGC was found in the N-terminal portion, while CCS was found in the C-terminal portion of the *HaTrxR1*, and these sequences were highly conserved among the selected insect species (Fig. 2A). Phylogenetic analysis showed that *HaTrxR1* was more closely related to the *BmTrxR1-X2* homologue (*B. mori*, XP_004921588.1) than other selected species, and this result was consistent with the evolutionary relationship predicted from the multiple alignment of amino acid sequences (Fig. 2B). The tertiary protein structure of *HaTrxR1* was constructed using the SWISS-MODEL server and PyMOL-v1.3r1 software, and the conserved redox active motifs (CVNVGC and CCS) were identified (Fig. 2C).

### Temporal and spatial expression profiles of *HaTrx2* and *HaTrxR1*

**Figure 3:**
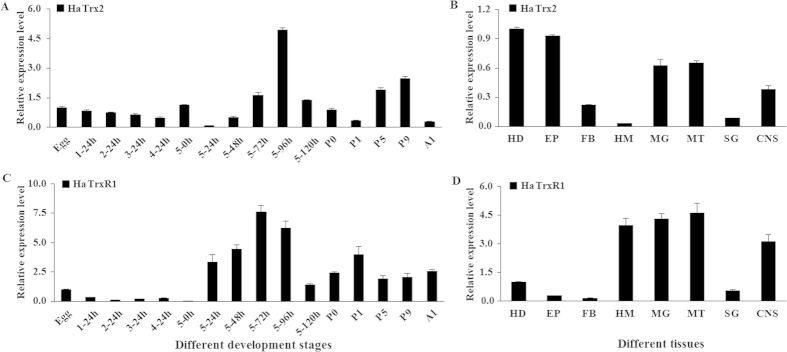
Expression profile of *HaTrx2* and *HaTrxR1* in different developmental stages and different larval tissues. (**A**) Transcript levels of *HaTrx2* during different developmental stages. 1–24h, 24 h larvae of 1st instar; 2–24 h, 24 h larvae of 2nd instar; 3–24 h, 24 h larvae of 3rd instar; 4–24 h, 24 h larvae of 4th instar; 5–0 h, 5–24 h, 5–48 h, 5–72 h, 5–96 h, and 5–120 h stand for 5th instar larvae at 0, 24, 48, 72, 96, and 120 h, respectively; P0, P1, P5, and P9 stand for 0, 1, 5, and 9 day pupae, respectively; A1, 1 day adults. The same as below. (**B**) Transcript levels of *HaTrx2* in the tissues of 5th instar 24 h larvae. HD, heads; EP, epidermis; FB, fat body; HM, hemolymph; MG, midgut; MT, malpighian tubule; SG, salivary glands; CNS, central nervous system. The same as below. (**C**) Transcript levels of *HaTrxR1* during different developmental stages. (**D**) Transcript levels of *HaTrxR1* in the tissues of 5th instar 24 h larvae. The data represent the mean ± standard deviation (SD) from 3 biological samples.

To determine the transcription profile of *HaTrx2* in different developmental stages and larval tissues in *H. armigera*, qRT-PCR was carried out using total RNA prepared from the above collected samples. Standard curves for the primers were generated before formal experiments. The correlation coefficients (*R*^*2*^) of the four genes (*HaTrx2*, *HaTrxR1*, *RPS15*, and *RPL32*) were greater than 0.99, and the amplification efficiencies of the primers were 98.06%, 103.97%, 105.20%, and 98.70%, respectively (Figure S1). The *HaTrx2* transcript showed ubiquitous expression in all developmental stages, mainly being expressed in the 96 h larvae of the 5th instar (Fig. 3A). The spatial expression profiles revealed that the *HaTrx2* gene could be detected in all of the investigated tissues, and the expression levels were higher in the head, epidermis, midgut and Malpighian tubules than other tissues (Fig. 3B).

The qRT-PCR results showed that *HaTrxR1* was mainly expressed in 24 h, 48 h, 72 h, and 96 h larvae of the 5th instar and the first-day pupae, with relatively lower expression being observed in other larval stages (Fig. 3C). The obtained spatial expression profiles showed that the *HaTrxR1* gene was mainly expressed in the hemocytes, midgut, Malpighian tubules, and CNS (Fig. 3D).

### The response of the expression profiles of *HaTrx2* and *HaTrxR1* to various types of adversity

**Figure 4:**
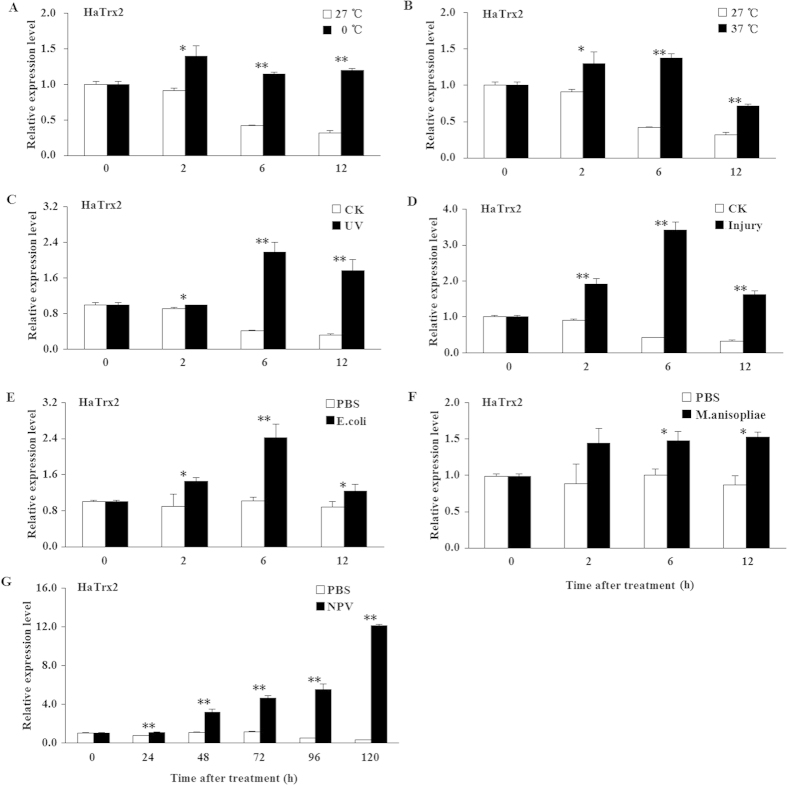
Expression profiles of *HaTrx2* under different abiotic and biotic stresses. Total RNA was harvested from *H. armigera* samples under different stress challenges, including low temperature (4 °C), high temperature (37 °C), UV light, mechanical injury, *E. coli*, *M. anisopliae*, and NPV infection, and then subjected to real-time PCR analysis. The data represent the mean ± SD of 3 biological samples. *0.01 < *P* < 0.05; **P < 0.01.

**Figure 5:**
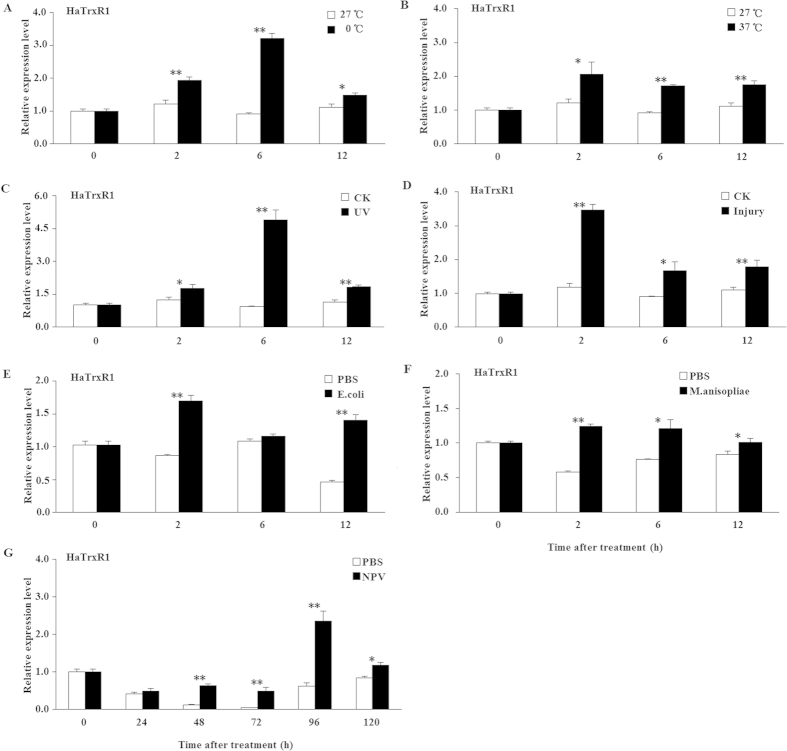
Expression profiles of *HaTrxR1* under different abiotic and biotic stresses. Total RNA was harvested from *H. armigera* samples under different stress challenges, including low temperature (4 °C), high temperature (37 °C), UV light, mechanical injury, *E. coli*, *M. anisopliae*, and NPV infection, and then subjected to real-time PCR analysis. The data represent the mean ± SD of 3 biological samples. *0.01 < *P *< 0.05; **P < 0.01.

To study the effect of various adverse stresses on *HaTrx2* and *HaTrxR1* transcription, larvae were challenged with low temperature, high temperature, UV light, mechanical injury, *E. coli* exposure, *M. anisopliae* exposure, and NPV infection. As shown in Fig. 4, the transcription of *HaTrx2* was significantly induced by the 0 °C, 37 °C, UV, mechanical injury, and *E. coli* exposure treatments at 2 h, 6 h, and 12 h, in addition to being increased at 6 h and 12 h after *M. anisopliae* exposure treatment and being markedly up-regulated at 24 h, 48 h, 72 h, 96 h, and 120 h after NPV infection. For the *HaTrxR1* transcript, we observed a similar tendency (Fig. 5). *HaTrxR1* transcription was markedly up-regulated at 2 h, 6 h, and 12 h after the 0 °C, 37 °C, UV, mechanical injury, *M. anisopliae* exposure treatments, in addition to being significantly up-regulated at 2 h and 12 h after *E. coli* exposure and being markedly increased at 48 h, 72 h, 96 h, and 120 h after NPV infection (Fig. 5). Taken together, all of the above results suggested that *HaTrx2* and *HaTrxR1* may play an important role in protection against the oxidative stress caused by various types of adversity, and especially NPV infection.

### ROS generation and lipid peroxidation in HaTrx2- or HaTrxR1-knockdown larvae and normal larvae

**Figure 6:**
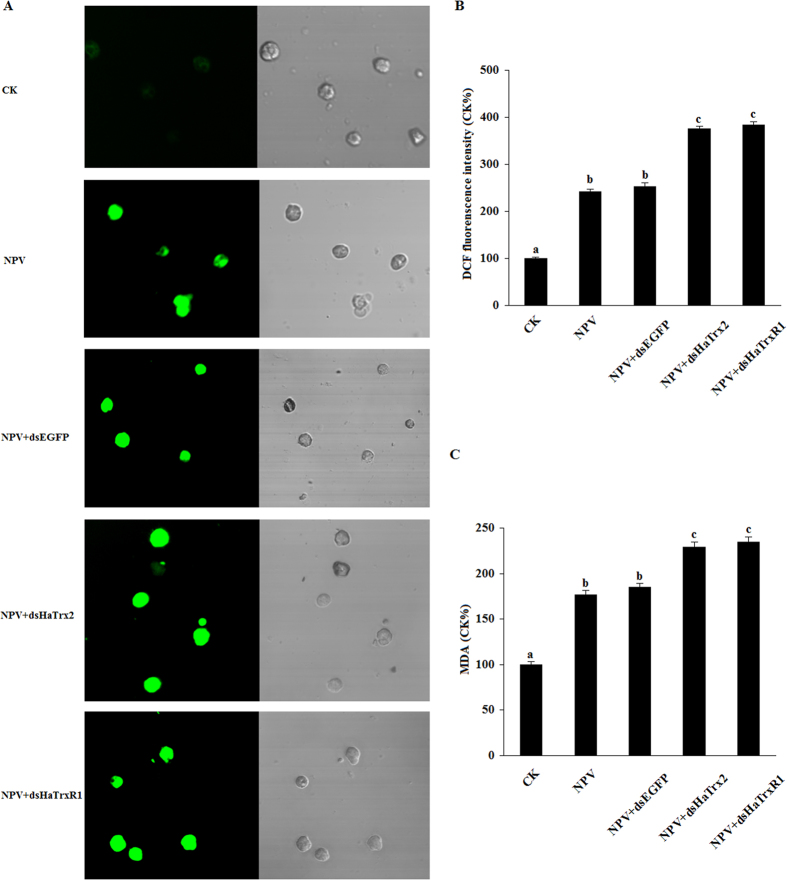
Stimulatory effect of HaTrx2- or HaTrxR1-knockdown on ROS levels in hemocytes of NPV infected *H. armigera* larvae. (**A**) Effects of HaTrx2- or HaTrxR1-knockdown on ROS generation in hemocytes. (**B**) The relevant DCF-fluorescent intensity quantification is shown as a percentage of the values found in larvae hemocytes of CK group. (**C**) The results of MDA generation and lipid peroxidation in *H. armigera* hemolymph after HaTrx2- or HaTrxR1-knockdown. The data represent the mean ± SD of 3 biological samples. a & b: signification difference, *P *< 0.05.

To confirm *HaTrx2* and *HaTrxR1* play vital roles in protecting *H. armigera* against oxidative damage caused by NPV infection, ROS generation was determined in HaTrx2- or HaTrxR1-knockdown larvae and normal larvae. As shown in Fig. 6A,B, the fluorescence intensity of larvae hemocytes in the NPV + dsHaTrx2 or NPV + dsHaTrxR1 group were both stronger than that in the NPV+dsEGFP or NPV groups.

As ROS damage also caused lipid peroxidation in living organism. We measured the concentration of a terminal product (malonyl dialdehyde, MDA) of lipid peroxidation in hemocytes after HaTrx2- or HaTrxR1-knockdown to confirm *HaTrx2* and *HaTrxR1* play vital roles in protecting *H. armigera* against oxidative damage caused by NPV infection. The results showed that MDA levels were markedly increased after HaTrx2- or HaTrxR1-knockdown compared to EGFP dsRNA injection or NPV infection (Fig. 6C).

### RNA interference and survival assay

To further confirm the functions of *HaTrx2* and *HaTrxR1*, the adverse stress of NPV infection was chosen because the expression of these two genes was increased to a greater extent by NPV infection than the other selected adverse stresses (Fig. 4 and 5).

**Figure 7:**
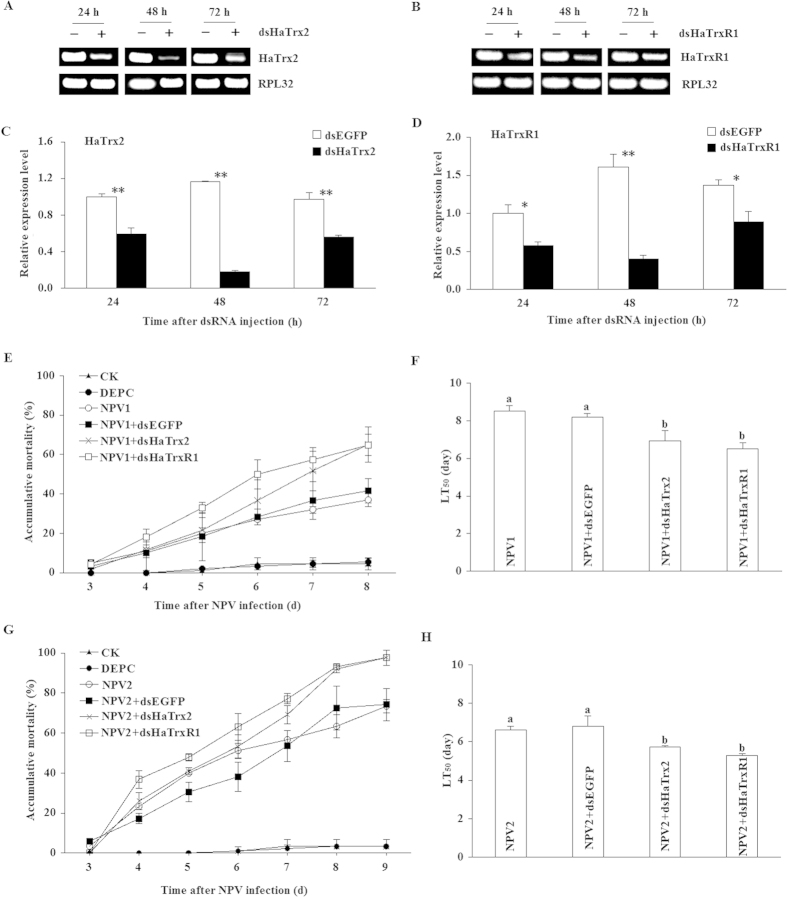
RNAi efficiency of *HaTrx2* or *HaTrxR1* dsRNA and effects of HaTrx2- or HaTrxR1-knockdown on the susceptibility of *H. armigera* larvae to NPV infection. (**A–D**) RNAi-induced reduction of *HaTrx2* and *HaTrxR1* transcription. First, 4th instar first-day larvae were fed with an artificial diet contaminated with 10 μL of NPV (1 × 10^6^ PIB/ml). At 48 h after NPV infection (at the concentration of 1 × 10^6^ PIB/ml or 1 × 10^7^ PIB/ml), a total of 15 μg of the dsRNA of *HaTrx2*, *HaTrxR1*, or *EGFP* was injected into each treatment larva. The samples of *H. armigera* larvae were collected 24, 48, and 72 h after dsRNA injection and then subjected to total RNA extraction, cDNA synthesis, and real-time PCR analysis. (**E**) Cumulative mortality of NPV infected larvae at the concentration of 1 × 10^6^ PIB/ml (NPV1) after HaTrx2- or HaTrxR1-knockdown. (**F**) LT_50_ values in the bioassay of NPV infected larvae at the concentration of 1 × 10^6^ PIB/ml after HaTrx2- or HaTrxR1-knockdown. (**G**) Cumulative mortality of NPV infected larvae at the concentration of 1 × 10^7^ PIB/ml (NPV2) after HaTrx2- or HaTrxR1-knockdown. (**H**) LT_50_ values in the bioassay of NPV infected larvae at the concentration of 1 × 10^7^ PIB/ml after HaTrx2- or HaTrxR1-knockdown. The data represent the mean ± SD of 3 biological samples. *0.01 < *P *< 0.05; **P < 0.01. a & b: signification difference, *P *< 0.05.

The results of agarose gel electrophoresis and real-time PCR analyses showed that the transcripts of *HaTrx2* and *HaTrxR1* were significantly decreased at 24 h, 48 h, and 72 h after *HaTrx2* and *HaTrxR1* dsRNA injection compared with EGFP dsRNA injection (Fig. 7A,B). *HaTrx2* expression was decreased by 40.77%, 84.43%, and 39.89% (Fig. 7C), while *HaTrxR1* expression was decreased 42.54%, 77.81%, and 35.06% (Fig. 7D) at 24 h, 48 h, and 72 h after the injection of *HaTrx2* or *HaTrxR1* dsRNA, respectively, compared with EGFP dsRNA injection.

qRT-PCR results also showed that expression level of *HaTrxR1* increased significantly after *HaTrx2* knockdown, however, *HaTrx2* expression level remained unchanged after *HaTrxR1* knockdown (Figure S2). To determine how is the NPV infection going on at 48 h post infection and after 48 h of dsRNA injection, qRT-PCR was used to quantify the virus gDNA abundance. As shown in Figure S3A, viral gDNA level at 48 h after NPV infection increased about 300 times than 0 h or 24 h after NPV infection. HaTrx2- or HaTrxR1-knockdown also obviously promoted viral gDNA levels compared to EGFP dsRNA injection at 48 h (Figure S3B).

At 48 h after NPV inoculation, *HaTrx2* dsRNA, *HaTrxR1* dsRNA, or EGFP dsRNA was injected into the larvae. The overall trend was that injection of *HaTrx2* dsRNA or *HaTrxR1* dsRNA accelerated the mortality of larvae infected with NPV at the both concentrations of 1.0 × 10^6^ PIB/mL (NPV1) and 1.0 × 10^7^ PIB/mL (NPV2) (Fig. 7E,G). When larvae infected with NPV at the concentration of 1.0 × 10^6^ PIB/mL, the LT_50_ (time required to reached 50% mortality) of NPV1 (8.54 d) and NPV1 + dsEGFP (8.20 d) larvae was significantly higher than in NPV1 + dsHaTrx2 (6.93 d) or NPV1 + dsHaTrxR1 (6.50 d) larvae (*P* < 0.05, Fig. 7F). When larvae infected with NPV at the concentration of 1.0 × 10^7^ PIB/mL, the LT_50_ of NPV2 (6.62 d) and NPV2 + dsEGFP (6.79 d) larvae was significantly higher than in NPV2 + dsHaTrx2 (5.73 d) or NPV2 + dsHaTrxR1 (5.27 d) larvae (*P* < 0.05, Fig. 7H). These results suggested that *HaTrx2* and *HaTrxR1* are required for resistance against NPV infection in *H. armigera* larvae.

## Discussion

Many studies addressing the functions of the thioredoxin system, which are involved in regulating cellular redox homeostasis and resisting oxidative stress caused by adversity, have been conducted in mammals^43^,^45^ and some model insect species^21^,^24^,^30^,^38^. However, research on Trx and TrxR in the lepidopteran pest *H. armigera* is lacking. In this study, *HaTrx2* and *HaTrxR1* were identified and characterized in the larvae of *H. armigera*. Sequence analysis suggested that *HaTrx2* shared high amino acid identity (61%–92%) with other insect counterparts, and all of these proteins contained the highly conserved CGPC active-site motif, which is essential for their catalytic activity^7^. Multiple alignment and phylogenetic analysis revealed that *HaTrxR1* shared 64%–83% sequence identity with other insect species, including the important active site sequence CVNVGC in the N-terminal portion and the CCS motif in the C-terminal extension (Fig. 2A)^30^. These results demonstrated that both *HaTrx2* and *HaTrxR1* possessed redox active sites and belonged to the typical Trx and TrxR families, respectively, and they might be involved in resistance to adversity.

The changes in *HaTrx2* and *HaTrxR1* transcription observed at different developmental stages showed that these two genes were mainly expressed in 5th instar and pupal stage. The obtained spatial expression profiles revealed that the *HaTrx2* gene was expressed at higher levels in the head, epidermis, midgut and Malpighian tubules than other tissues (Fig. 3B), suggested that it may play vital roles in antioxidant defense in these tissues, which are central organs in metabolism and detoxification. The expression of Trx in larval tissues appears to show a species-dependent pattern: *BmTrx* is mainly expressed in the fat body and silk gland^21^; *AccTrx1* and *AccTrx-like1* exhibit higher expression in the epidermis than in other tissues^23^,^25^; and *AccTrx2* is expressed at higher levels in the brain and midgut^24^. However, the *HaTrxR* gene is mainly expressed in the hemocytes, midgut, Malpighian tubules, and CNS (Fig. 3D), implying that it may mainly play crucial roles in these tissues with antioxidant functions.

It has been reported that adverse environmental factors, such as pesticides, heavy metals, UV radiation, and abnormal temperatures can lead to oxidative damage to living organisms^46^. A majority of antioxidant enzymes, such as peroxidases and catalases, play significant roles in the scavenging or quenching of oxidants and, thus, constitute a primary short-term line of defense. In previous studies, the expression of *BmTrx* in the fat body of *B. mori* larvae was shown to be greatly increased after treatment with H_2_O_2_, paraquat, low or high temperatures, or microorganism (bacterium, fungus, and NPV) infection^21^; *AccTrx-like1* was found to be up-regulated by treatment with H_2_O_2_ or temperatures of 4, 15, and 25 °C^23^; *AccTrx2* was shown to be stimulated by treatment with H_2_O_2_, temperatures of 4, 16, and 25 °C, acaricide, cyhalothrin, phoxim, paraquat, and HgCl_2_^24^; and *AccTrx1* was found to be induced by treatment with H_2_O_2_, temperatures of 4, 16, and 42 °C and pesticides (acaricide, phoxim, cyhalothrin, and paraquat)^25^, suggesting that Trx may play important roles in protection against oxidative stress caused by an adverse environment. The TrxR gene of *A. gambiae* was also shown to be induced by injury, bacterial challenge, and malaria infection^47^. In the present study, the transcripts of *HaTrx2* and *HaTrxR1* were significantly induced by various types of adversity, including low temperature, high temperature, UV light, mechanical injury, *E. coli* exposure, *M. anisopliae* exposure, and NPV infection, which suggests that *HaTrx2* and *HaTrxR1* may participate in resistance to these adverse conditions. The possible mechanism underlying Trx and TrxR involvement in antioxidant defense may be elucidated as follows: ROS are first formed under adverse stress and then act on cellular biomacromolecules that are susceptible to oxidation stress by disrupting intracellular redox homeostasis, and Trx and TrxR may be play crucial roles in the removal of excessive ROS to protect organisms^48^.

In this study, to confirm the role of *HaTrx2* and *HaTrxR1* in the removal of excessive ROS caused by NPV infection to protect *H. armigera* larvae, the expression of *HaTrx2* and *HaTrxR1* was successfully knockdown with the injection of the related dsRNA, as examined by semi-quantitative RT-PCR and qRT-PCR. Further study confirmed that HaTrx2- or HaTrxR1-knockdown increased ROS production in hemocytes and also increased the lipid damage in NPV infected *H. armigera* larvae. Together, these results indicated that *HaTrx2* and *HaTrxR1* may participate in the removal of excessive ROS caused by NPV infection in *H. armigera*. However, further study to provide in-depth confirmation is warranted.

In *S. litura*, larval mortality was accelerated after knockdown of the antioxidant gene *SlTpx* through dsRNA interference in the presence of *N. rileyi* infection, suggesting that *SlTpx* plays a vital role in resisting oxidative damage caused by *N. rileyi* infection^44^. In *D. melanogaster*, *Drosophila* cells become susceptible to H_2_O_2_ treatment after knockdown of the Tpx transcript through RNAi^49^. Here, the expression of *HaTrx2* and *HaTrxR1* was found to be significantly stimulated by NPV infection, which is widely applied in the management of the pest *H. armigera* due to its strong pathogenicity. In a further experiment, knockdown of *HaTrx2* or *HaTrxR1* transcripts resulted in increased sensitivity to NPV infection and shortened LT_50_ values. All of these observations indicated that expression of *HaTrx2* and *HaTrxR1* is essential in defense against NPV infection in *H. armigera* larvae.

In conclusion, we have characterized two typical thioredoxin system genes from *H. armigera*, and determined the temporal and spatial expression profiles of *HaTrx2* and *HaTrxR1*. The transcription of *HaTrx2* and *HaTrxR1* was induced by various types of adversity (low temperature, high temperature, UV light, mechanical injury, *E. coli* exposure, *M. anisopliae* exposure, and NPV infection), suggesting *HaTrx2* and *HaTrxR1* play important roles in resistance to various types of adversity. HaTrx2- or HaTrxR1-knockdown increased ROS production in hemocytes and also increased the lipid damage in NPV infected *H. armigera* larvae. RNAi experiments further confirmed that *HaTrx2* and *HaTrxR1* are involved in resistance to NPV infection. These observations provide powerful evidence demonstrating that *HaTrx2* and *HaTrxR1* play vital roles in protecting *H. armigera* against oxidative damage and enrich our knowledge of the thioredoxin system in insects. Therefore, the development of novel chemicals and microbial pesticides targeting *HaTrx2* or *HaTrxR1* for *H. armigera* control will require further in-depth research.

## Methods

### Insect maintenance and *H. armigera* NPV

*H. armigera* were reared in our laboratory with an artificial diet^50^ at a constant temperature of 26 ± 1 °C, under 75 ± 10% RH and a 16 L: 8 D light regime. The larvae were individually reared in separate glass tubes (5.5 cm in length × 2.0 cm in diameter) after the 3rd instar stage to prevent cannibalism.

Raw powder of *H. armigera* NPV (5 × 10^11^ PIB/g) was bought from the Henan Jiyuan Baiyun Industry Co., Ltd (China) and stored at 4 °C for later use.

### Sequence analysis of *HaTrx2* and *HaTrxR1*

The GenBank accession number of *HaTrx2* and *HaTrxR1* were JQ744277.1 and KM658552. The physicochemical properties of *HaTrx2* and *HaTrxR1* were analyzed using the online bioinformatics ProtParam tool (http://web.expasy.org/protparam/). Homologous protein sequences of Trxs and TrxRs from various species were obtained from the NCBI database and aligned using DNAman6.0.3 software. Phylogenetic analysis was carried out using MEGA5.10 software. Finally, the tertiary protein structures of *HaTrx2* and *HaTrxR1* were predicted with the online server SWISS-MODEL and were modified with PyMOL-v1.3r1 software^51^.

### Developmental analysis and tissue distribution of *HaTrx2* and *HaTrxR1*

To examine the temporal expression profiles of *HaTrx2* and *HaTrxR1*, *H. armigera* samples were collected at different developmental stages, including eggs; 24 h larvae of the first, second, third, and 4th instar; 0, 24, 48, 72, 96, and 120 h larvae of the 5th instar; 0, 1, 3, 5 and 9 day pupae; and 1 day adults (equal numbers of females and males). To analyze the spatial expression patterns of *HaTrx2* and *HaTrxR1*, the tissues of the 5th instar 48 h larvae were collected, including the head, epidermis, fat body, hemocytes, midgut, Malpighian tubules, salivary glands, and central nervous system (CNS)^52^. Each sample was repeated three times and immediately stored at −80 °C for total RNA extraction.

### Effect of different types of stress on the expression of *HaTrx2* and *HaTrxR1*

For the temperature treatments, 0 °C (low temperature) and 37 °C (high temperature) were chosen^21^,^53^. The first-day larvae of the 5th instar were held for 12 h under the two temperatures, while the controls were maintained at 27 °C (normal temperature)^21^. In the UV treatment, the first-day larvae of the 5th instar were irradiated with 300 nm wavelength light, and the control larvae were kept under normal light for 12 h^53^. In the mechanical injury experiment, each larva was impaled 10 times with an insect pin (30 × 0.5 mm), and normal larvae were used as controls. In the *E. coli* infection treatment, *E. coli* cells were diluted in PBS and subsequently injected into the abdomens of first-day larvae of the 5th instar with a syringe, injecting 10 μL of 1.0 × 10^5^ *E. coli* cells per larva^21^. Control larvae were injected with an equal volume of PBS (10 μL/larva). For challenge by *M. anisopliae*, *M. anisopliae* was first inoculated on potato dextrose agar plates and incubated at 26 °C for 7–10 days. The produced conidia were then scraped and diluted with sterile water containing 0.1% Tween−80 to 1.9 × 10^8^ conidia/μL, which has been reported as the LC_50_ concentration of *H. armigera*^54^. The first-day larvae of the 5th instar were injected with 5 μL of the diluted *M. anisopliae* suspension, and control larvae were injected with an equal volume of PBS (5 μL/larva)^55^. The treatment and control larvae from each group were collected at 0, 2, 6, and 12 h after treatment, then immediately stored at −80 °C for further total RNA extraction. For the virus challenge, the first-day larvae of the 4th instar were inoculated with 10 μL of NPV at a concentration of 1.0 × 10^6^ PIB/mL per larva, and control larvae were inoculated with 10 μL of sterile water. The treatment and control larvae were collected after 0, 24, 48, 72, 96, and 120 h and then immediately stored at −80 °C for later total RNA extraction^46^. At least three independent biological replications were carried out in each of the adverse condition experiments, and at least 15 larvae were used in both the control and treatment replications.

### Primer design

The primers of *HaTrx2* and *HaTrxR1* used for RT-PCR, real-time PCR, and dsRNA synthesis were designed with DNAClub software according to their sequences. The *H. armigera* ribosomal proteins S15 (*RPS15*) and L32 (*RPL32*) were used as internal controls for real-time PCR normalization. All of the primers were synthesized by Sangon Biotechnology Co., Ltd. (Shanghai, China) (Table 1).

### Total RNA extraction, cDNA synthesis, and real-time PCR amplification

Total RNA was extracted from the above samples using the TRIzol reagent (Invitrogen, USA) following the manufacturer’s protocols. The purity and concentration of the RNA samples were determined three times with an ultraviolet spectrophotometer (Abs260) to reduce deviation. First-strand complementary DNA (cDNA) was synthesized from 1 μg of total RNA following the instruction manual of the PrimeScript RT reagent kit with gDNA Eraser (Takara, Kyoto, Japan) and immediately stored at −80 °C for later use. The cDNA samples were evaluated in triplicate.

qRT-PCR was performed using SYBR green supermix (TaKaRa) in a Bio-Rad CFX Connect^TM^ Real-Time PCR System (Bio-Rad, USA) to determine the gene expression levels. The real-time PCR amplification conditions for *HaTrx2*, *HaTrxR1*, *RPS15*, and *RPL32* are listed in Table S1. The reliability of the qRT-PCR results was confirmed through standard curve and melting curve analyses. Standard curves were generated using 10-fold dilution series of cDNA as a template for each treatment, employing a linear regression model (Figure S1s)^56^. The efficiencies (E) of the primers used for qRT-PCR were calculated according to the equation: E = (10^[−1/slope]^−1) × 100%^57^. The specificity of the amplified product was further confirmed through melting curve analysis from 65 °C to 95 °C and agarose gel electrophoresis. The mRNA expression of target genes was quantified using the comparative CT (cross threshold) method^58^. The CT value of the reference gene was subtracted from the CT value of the target gene to obtain ΔCT. The normalized fold changes of target gene mRNA expression were expressed as 2^−ΔΔCT^, where ΔΔCT is equal to ΔCT_treated sample_ –ΔCT_control_.

### Synthesis of dsRNA and detecting of RNAi efficiency

To synthesize the dsRNAs, gene-specific primers containing a T7 polymerase promoter sequence were used to amplify the target sequences via reverse transcription-PCR (RT-PCR) (Table 1). The applied RT-PCR amplification conditions are listed in Table S1. The MEGAscript RNAi kit (Ambion) was employed to synthesize the dsRNAs according to the manufacturer’s instructions. DNase and ribonuclease (RNase) were used to remove the template DNA and single-stranded RNA from the transcription reaction. dsRNAs were purified with MEGAclear columns (Ambion) and eluted with diethyl pyrocarbonate (DEPC)-treated nuclease-free water. The purity and concentration of dsRNA were then measured via ultraviolet spectrophotometry (Table S2) and gel electrophoresis. As a negative control, dsRNA of enhanced green fluorescent protein (*EGFP*) was also synthesized.

To evaluate the effects of RNAi on the gene expression, the first day larvae of 4th instar which inoculated with NPV (total quantity of 10^4^ PIB per larva) at 48 h were injected with 15 μg dsRNA of *HaTrx2*, *HaTrxR1*, or EGFP, respectively. The whole-body samples were collected at 24, 48, and 72 h after dsRNA injection, and then used for total RNA extraction and real-time PCR analysis. To determine the relation between *HaTrx2* and *HaTrxR1*, *HaTrxR1* expression was measured after *HaTrx2* dsRNA injection at 48 h and *HaTrx2* expression was also measured after *HaTrxR1* dsRNA injection at 48 h.

### Measurement of ROS production and lipid peroxidation

To study the effect of RNAi on ROS generation in hemocytes, newly molted 4th instar larvae were fed the NPV-contaminated diet (10 μL of NPV at the concentration of 1.0 × 10^6^ PIB/mL per larva) as the method of NPV challenge. After NPV infection 48 h, the infected larvae were injected with the dsRNA of *EGFP*, *HaTrx2*, or *HaTrxR1*, respectively. After dsRNA injection 48 h, hemolymph of each groups were collected from *H. armigera* and centrifuged immediately at 4000 × g at 4 °C for 10 min to isolate hemocytes. Then, ROS production was measured with Reactive Oxygen Species Assay Kit (Nanjing Jiancheng Bioengineering Institute, Nanjing, China). Hemocytes were incubated with DCFH-DA (2, 7-dichlorofuorescin diacetate) at a final concentration of 10 μM for 20 min. Hemocyte morphology was observed using a OLYMPUS BX61 (Olympus, Tokyo, Japan) laser scanning confocal microscope. ROS production in hemocytes was measured fluorometrically at excitation and emission wavelengths of 488 and 525 nm, respectively.

Usually, ROS damage caused lipid peroxidation in the organism. As a terminal product of lipid peroxidation, MDA was measured to evaluate the degree of lipid peroxidation in the hemolymph using MDA Assay Kit (Nanjing Jiancheng Bioengineering Institute, Nanjing, China).

### qRT-PCR analysis of virus gDNA abundance at 48 h post NPV infection and after 48 h of dsRNA injection

qRT-PCR was used to quantify the virus abundance in NPV infected larvae at 48 h post NPV infection and after 48 h of dsRNA injection using specific primers to the polyhedrin gene (Accession no. NC_002654.2) of *HaSNPV*. The first-day larvae of the 4th instar were inoculated with 10 μL of NPV at a concentration of 1.0 × 10^6^ PIB/mL per larva as the above method. The samples of whole body were collected at 0, 24, and 48 h after NPV infection, respectively. After 48 h of NPV infection, dsRNA of *EGFP*, *HaTrx2*, or *HaTrxR1* were injected to the NPV infected larvae, respectively. The sample of each treatment were collected at 48 h after dsRNA injection. The genomic DNA was extracted and used in qRT-PCR technique as described^59^. *H. armigera* actin gene (Accession no. HM629437.1) was used as the housekeeping gene for normalization the data of virus gDNA quantification.

### RNA interference and survival assay

To determine the effects of HaTrx2- or HaTrxR1-knockdown on the susceptibility of *H. armigera* larvae to NPV infection, two inoculation doses (10 μL of NPV at the concentration of 1.0 × 10^6^ PIB/mL or 1.0 × 10^7^ PIB/mL per larva) were selected^60^.

The first-day larvae of the 4th instar were inoculated with 10 μL of NPV at a concentration of 1.0 × 10^6^ PIB/mL per larva (the group of “NPV1”) according to the above method (for the virus challenge). Control larvae were inoculated with 10 μL of sterile water (the “CK” group) or injected with 10 μL DEPC solution (the “DEPC” group). At 48 h after NPV inoculation, 15 μg of *HaTrx2* dsRNA (the “NPV1 + dsHaTrx2” group), 15 μg of *HaTrxR1* dsRNA (the “NPV1 + dsHaTrxR1” group), or 15 μg of EGFP dsRNA (the “NPV1 + dsEGFP” group) was injected into the proleg of each *H. armigera* larva using a capillary microsyringe. As the above method, The first-day larvae of the 4th instar were inoculated with 10 μL of NPV at a concentration of 1.0 × 10^7^ PIB/mL per larva (the group of “NPV2”). At 48 h after NPV infection, 15 μg of *HaTrx2* dsRNA (the “NPV2 + dsHaTrx2” group), 15 μg of *HaTrxR1* dsRNA (the “NPV2 + dsHaTrxR1” group), or 15 μg of EGFP dsRNA (the “NPV2 + dsEGFP” group) was injected into the proleg of each *H. armigera* larva. The number of dead larvae was observed and recorded in each group until the larvae pupated. At least 30 larvae were included in each replicate, and every treatment was replicated three times.

### Statistical analysis

The real-time PCR experiments and RNAi experiments were both carried out with three independent replications, and the results are presented as the means ± standard deviation (SD). Statistically significant differences in gene expression observed in the real-time PCR assays are denoted by *(0.01 < *p* < 0.05) and **(*p* < 0.01), as obtained through pair-wise Student’s *t-test* analysis. The mortality rate was analyzed using ANOVA followed by Turkey’s HSD multiple comparison test in SPSS 17.0 software to detect statistically significant differences between different groups (*p* < 0.05).

## Additional Information

**How to cite this article**: Zhang, S. *et al.* Sequence analysis, expression profiles and function of thioredoxin 2 and thioredoxin reductase 1 in resistance to nucleopolyhedrovirus in *Helicoverpa* armigera. *Sci. Rep.* **5**, 15531; doi: 10.1038/srep15531 (2015).

## Supplementary Material

Supplementary Information

## Figures and Tables

**Table 1:** Primers used in the current study.

Gene name (Abbreviation)	Description	Sequence (5′–3′)	Product length (bp)
HaTrx2-qF	Real-time PCR primer, forward	GTCGATCCACATCAAGGAC	94
HaTrx2-qR	Real-time PCR primer, reverse	GCACCAAGTGGCCATGAAG	
HaTrx2-RNAiF1	The first step PCR primer of dsRNA synthesis, forward	GGACCCTGCAAGATGATCG	213
HaTrx2-RNAiR1	The first step PCR primer of dsRNA synthesis, reverse	CAGGATAGTGTTCCTCAG	
HaTrx2-RNAiF2	The second step PCR primer of dsRNA synthesis, forward	GATCACTAATACGACTCACTATAGGGAGAGGACCCTGCAAGATGATCG	271
HaTrx2-RNAiR2	The second step PCR primer of dsRNA synthesis, reverse	GATCACTAATACGACTCACTATAGGGAGACAGGATAGTGTTCCTCAG	
HaTrxR1-qF	Real-time PCR primer, forward	CGAGGTCATACAGGGCTTC	125
HaTrxR1-qR	Real-time PCR primer, reverse	TGCGCTTGGTGATGTTGAGG	
HaTrxR1-RNAiF1	The first step PCR primer of dsRNA synthesis, forward	GATGCTCATACGATCATCGC	391
HaTrxR1-RNAiR1	The first step PCR primer of dsRNA synthesis, reverse	CCTTGAGCTGTCCTGACTC	
HaTrxR1-RNAiF2	The second step PCR primer of dsRNA synthesis, forward	GATCACTAATACGACTCACTATAGGGAGAGATGCTCATACGATCATCGC	449
HaTrxR1-RNAiR2	The second step PCR primer of dsRNA synthesis, reverse	GATCACTAATACGACTCACTATAGGGAGACCTTGAGCTGTCCTGACTC	
EGFP-RNAiF1	The first step PCR primer of dsRNA synthesis, forward	CCTGAAGTTCATCTGCACCAC	538
EGFP-RNAiR1	The first step PCR primer of dsRNA synthesis, reverse	CTCCAGCAGGACCATGTGATC	
EGFP-RNAiF2	The second step PCR primer of dsRNA synthesis, forward	GATCACTAATACGACTCACTATAGGGAGACCTGAAGTTCATCTGCACCAC	596
EGFP-RNAiR2	The second step PCR primer of dsRNA synthesis, reverse	GATCACTAATACGACTCACTATAGGGAGACTCCAGCAGGACCATGTGATC	
Poly-qF	Real-time PCR primer, forward	CAAACCGAACCGTTGTTACC	171
Poly-qR	Real-time PCR primer, reverse	TGCAAGTTCATAACGGGAC	
ACT-qF	Real-time PCR primer, forward	GACGGTCAGGTCATCACCATC	151
ACT-qR	Real-time PCR primer, reverse	ACAGGTCCTTACGGATGTCA	
RPS15-qF	Real-time PCR primer, forward	CTGAGGTCGATGAAACTCTC	107
RPS15-qR	Real-time PCR primer, reverse	CTCCATGAGTTGCTCATTG	
RPL32-qF	Real-time PCR primer, forward	CATCAATCGGATCGCTATG	152
RPL32-qR	Real-time PCR primer, reverse	CCATTGGGTAGCATGTGAC	
