# Supplementary material for: Sequence analysis, expression profiles and function of thioredoxin 2 and thioredoxin reductase 1 in resistance to nucleopolyhedrovirus in Helicoverpa armigera
Source: Sci Rep. 2015 Oct 27;5:15531. doi: 10.1038/srep15531 (PMC4621414; doi:10.1038/srep15531)
Supplement: Supplementary Information [file srep15531-s1.doc]

**Sequence analysis, expression profiles and function of thioredoxin 2 and thioredoxin reductase 1 in resistance to nucleopolyhedrovirus in *Helicoverpa armigera***

Songdou Zhang^1^, Zhen Li^1^, Xiaoge Nian^1^, Fengming Wu^1^, Zhongjian Shen^1^, Boyu Zhang^1^, Qingwen Zhang^1^ & Xiaoxia Liu^1^,*

^1^Department of Entomology, China Agricultural University, Beijing, 100193, China

*Corresponding author: Dr. Xiaoxia Liu, Phone: +86 10 62733946, Fax: +86 10 62733946, E-mail: liuxiaoxia611@cau.edu.cn.

**Supporting Information Captions**

Figure S1. The standard curves and correlation coefficients (*R^2^*) of the four genes *HaTrx2*, *HaTrxR1*, *RPS15*, and *RPL32*.

Figure S2. Effect of *HaTrx2* knockdown on *HaTrxR1* expression and *HaTrxR1* knockdown on *HaTrx2* expression.

Figure S3. qRT-PCR analysis of virus gDNA abundance at 48 h post NPV infection and after 48 h of dsRNA injection.

Table S1. qRT-PCR and RT-PCR amplification conditions.

Table S2. The concentration and purity of dsRNA.


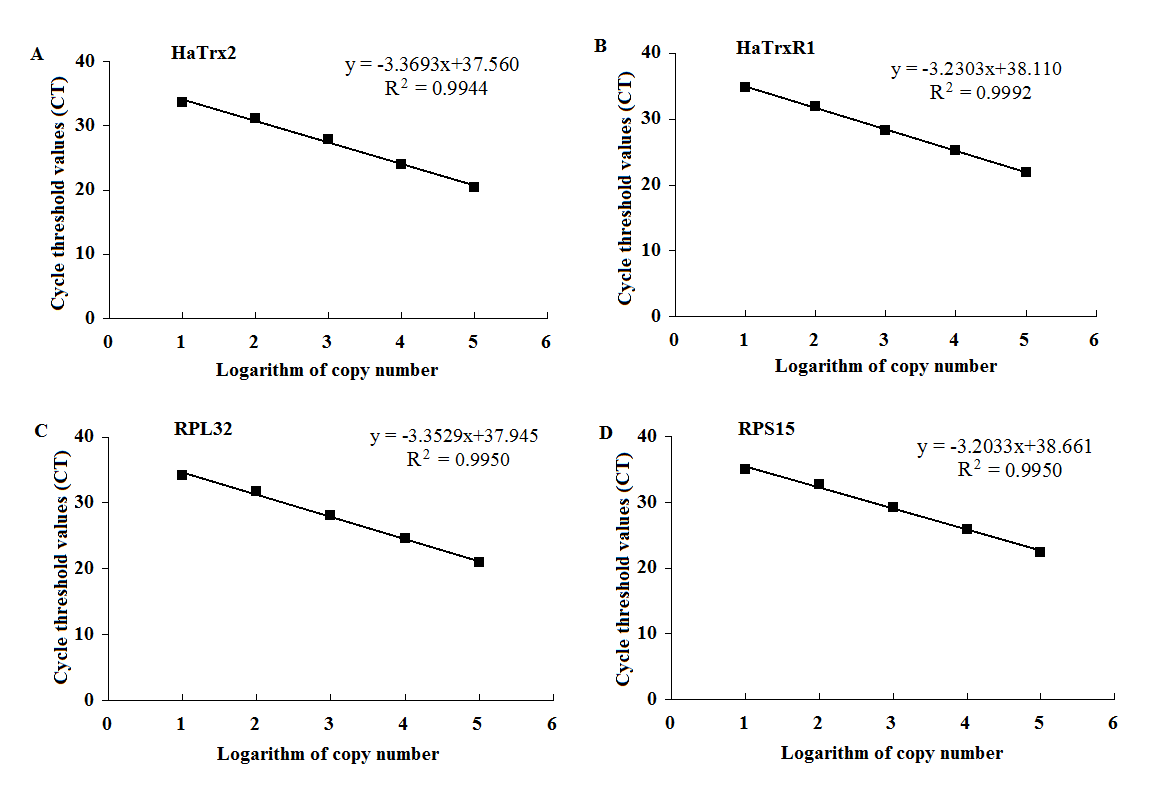


**Figure S1. The standard curves and correlation coefficients (*R^2^*) of the four genes *HaTrx2*, *HaTrxR1*, *RPS15*, and *RPL32*.**

**
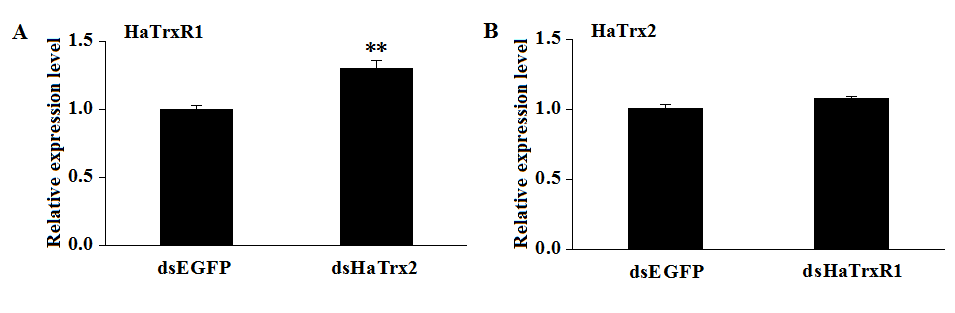
**

**Figure S2**. **Effect of *HaTrx2* knockdown on *HaTrxR1* expression and *HaTrxR1* knockdown on *HaTrx2* expression.**

(A) *HaTrxR1* expression levels after *HaTrx2* knockdown. (B) *HaTrx2* expression levels after *HaTrxR1* knockdown. First, 15 μg/larva of dsRNA of *HaTrx2*, *HaTrxR1*, or *EGFP* was injected into the first-day larvae of 4th instar in each treatment. The samples of *H. armigera* larvae were collected at 48 h after dsRNA injection and then subjected to total RNA extraction, cDNA synthesis, and real-time PCR analysis. The data represent the mean ± SD of 3 biological samples. *0.01 < *P* < 0.05; ***P* < 0.01.


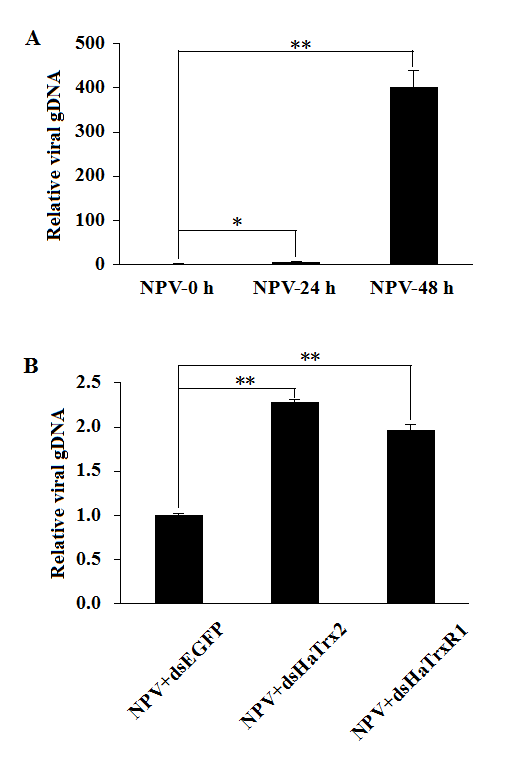


**Figure S3**. **qRT-PCR analysis of virus gDNA abundance at 48 h post NPV infection and after 48 h of dsRNA injection.**

(A) qRT-PCR analysis of virus gDNA abundance at 48 h post NPV infection. (B) qRT-PCR analysis of virus gDNA abundance after 48 h of *EGFP*, *HaTrx2*, or *HaTrxR1* dsRNA injection. Quantification of viral gDNA abundance in each treatment by qRT-PCR using specific primers to the NPV *polyhedrin* gene. The data represent the mean ± SD of 3 biological samples. *0.01 < *P* < 0.05; ***P* < 0.01.

**Table S1. qRT-PCR and RT-PCR amplification conditions**.

| Primer pairs | Amplification conditions |
| --- | --- |
| HaTrx2-qF/HaTrx2-qR | 95 °C for 30 sec, followed by 40 cycles of 95 °C for 10 sec and 60 °C for 30 sec |
| HaTrx2-RNAiF1/HaTrx2-RNAiR1 | 94 °C for 3 min, followed by 35 cycles of 94 °C for 45 sec, 54 °C for 45 sec, and 72 °C for 45 sec, and a final elongation at 72 °C for 10 min |
| HaTrx2-RNAiF2/HaTrx2-RNAiR2 | 94 °C for 3 min, followed by 40 cycles of 94 °C for 1 min, 56 °C for 1 min, and 72 °C for 1 min, and a final elongation at 72 °C for 10 min |
| HaTrxR1-qF/HaTrxR1-qR | 95 °C for 30 sec, followed by 40 cycles of 95 °C for 10 sec and 60 °C for 30 sec |
| HaTrxR1-RNAiF1/HaTrxR1-RNAiR1 | 94 °C for 3 min, followed by 35 cycles of 94 °C for 45 sec, 54 °C for 45 sec, and 72 °C for 45 sec, and a final elongation at 72 °C for 10 min |
| HaTrxR1-RNAiF2/HaTrxR1-RNAiR2 | 94 °C for 3 min, followed by 40 cycles of 94 °C for 1 min, 56 °C for 1 min, and 72 °C for 1 min, and a final elongation at 72 °C for 10 min |
| EGFP-RNAiF1/EGFP-RNAiR1 | 94 °C for 3 min, followed by 35 cycles of 94 °C for 45 sec, 54 °C for 45 sec, and 72 °C for 45 sec, and a final elongation at 72 °C for 10 min |
| EGFP-RNAiF2/EGFP-RNAiR2 | 94 °C for 3 min, followed by 40 cycles of 94 °C for 1 min, 56 °C for 1 min, and 72 °C for 1 min, and a final elongation at 72 °C for 10 min |
| Poly-qF/Poly-qR | 95 °C for 30 sec, followed by 40 cycles of 95 °C for 10 sec and 60 °C for 30 sec |
| ACT-qF/ACT-qR | 95 °C for 30 sec, followed by 40 cycles of 95 °C for 10 sec and 60 °C for 30 sec |
| RPS15-qF/RPS15-qR | 95 °C for 30 sec, followed by 40 cycles of 95 °C for 10 sec and 60 °C for 30 sec |
| RPL32-qF/RPL32-qR | 95 °C for 30 sec, followed by 40 cycles of 95 °C for 10 sec and 60 °C for 30 sec |

**Table S2. The concentration and purity of dsRNA**.

| Gene name | Concentration (ng/μL) | A260/A280 |
| --- | --- | --- |
| dsHaTrx2 | 3854.92 | 1.86 |
| dsHaTrxR1 | 3424.57 | 1.94 |
| dsEGFP | 3571.21 | 2.02 |
